# The Book World of Medicine and Science

**Published:** 1904-02-06

**Authors:** 


### THE BOOK WORLD OF MEDICINE AND SCIENCE.

THE PURIN BODIES OF FOOD-STUFFS AND THE RÔLE OF URIC ACID IN HEALTH AND DISEASE. By J. WALKER HALL, M.D., Assistant Lecturer and Demonstrator in Pathology, Owens College, etc. (Second edition (revised). London and Manchester: Sherratt and Hughes. 1903. Price 4s. 6d. net.)

THIS concise and well-arranged monograph presents the results of a well-directed investigation into the action of purin bodies upon the system, and discusses the evidence as to their metabolism. It is well known that purins ingested in the food, even though in small quantities, exert a very definite action upon the body, and Dr. Hall has investigated by means of a series of experiments what is the normal amount of purin existing in different food-stuffs, and their action on the various systems of the body. He has devised a new and very convenient method for the estimation of purin bodies in the animal tissues which is not open to the many objections of the older procedures. Feeding experiments were conducted on different subjects with purin bodies, and the results obtained are detailed and go to show that the urinary purin bodies excreted, mostly as uric acid, corresponded to about 50 to 60 per cent. of the purin bodies ingested in the food. The rapidity of purin metabolism was found to be more rapid than hitherto thought. The "free" purins of the food when ingested are very quickly oxidised and decomposed, the whole of the hypoxanthin nitrogen being voided in four hours, and that of the uric acid in eight hours. This, however, does not include the "bound" purins or nucleins of the food, the excretion of which was spread over one or two days. Feeding experiments were also made on rabbits: they were given a much larger amount of purin in their food than they were accustomed to, the result being hindered growth, and very marked degenerative cell changes in the liver, and to a less extent in the kidneys. The origin and fate of the endogenous purins, *i.e.*, those formed entirely by the metabolism of the body, are also subjected to discussion, and Dr. Hall inclines to the view that although the leucocytes aid in their formation, such are chiefly due to the cell changes which result in the maintenance of bodily functions. Uric acid is shown to be a necessary result of normal nuclein metabolism. In disease it is often symptomatic of conditions which hinder or prevent its solubility and excretion and does not itself cause the lesions which accompany uric-acidæmia. Results of the administration of drugs show that these are useless to increase the solubility of uric acid in the blood-stream, though they may do good in promoting the normal processes of nuclein metabolism through hepatic stimulation. The general conclusions of Dr. Hall's researches point to the need for determinations of the endogenous purins in many diseases either by the use of purin-free food, or by the aid of tables giving the percentages of purins in food stuffs, and the necessary calculations therefrom. In order to assist the clinician, Dr. Hall has wisely introduced his purinometer, and fully describes the method of its use. The whole book is written in a clear and concise manner, and is one which should be of value in the hands of those interested in chemical physiology and clinical pathology. There is an excellent bibliography, a useful appendix containing descriptions of methods, and a good index.

A SHORT PRACTICE OF GYNÆCOLOGY. By HENRY JELLETT. (London: J. and A. Churchill. 398 Pages. 223 Illustrations. Price 10s. 6d.)

THE author tells us in the preface that in its essentials the practice taught in this book is the practice of the Rotunda Hospital. Regarded from this point of view it forms an interesting review of the opinions and practice of one school of gynæcologists; but it contains too many inaccuracies, and its pathology is of too unscientific a nature to allow it to take a high place amongst text-books for students. For instance, we read that the uterus is lined with cubical epithelium, that this epithelium together with portions of the stroma and glandular epithelium are shed during menstruation, that kinks of the uterus and narrowness of the cervical canal are common causes of dysmennorhœa, and that "reflex menorrhagia" may be caused by falls upon the breech and the too frequent use of hot baths. These and many other examples we could quote show a lack of appreciation of the recent advances in pathology and physiology. The book is well and properly illustrated, and the section, consisting of over 160 pages, which deals with gynæcological operations is excellent.

GUY'S HOSPITAL, 1724–1902: A TRIBUTE TO ITS FOUNDER AND A RECORD OF ITS WORK. Illustrated. (London: Ash and Co., Ltd. 1903. Price 2s. 6d.)

THE learned history of Guy's has been written by Sir Samuel Wilks and the late Mr. G. T. Bettany. This is a popular sketch of the progress of the institution during its long existence, designed to show, by the help of pictures, what it was originally and what it has grown to be, but without recapitulating from the larger work those developments of medical science in which it has borne a distinguished part, or the lives of its distinguished physicians and surgeons. It gives a graphic view of the everyday life of a great hospital, and affords proof that Guy's has always been alive to the need of adaptation to new circumstances. What has been said so often in general terms of the increasing expenses of hospital treatment is illustrated here in a particular case, and in such a manner as to show both that benefactions are needed and how they are spent.

THE ADVERTISERS' A. B. C. 1904. (Publishers: Messrs. T. B. Browne, 163 Queen Victoria Street, E.C. Price 10s. 6d.)

THIS handsome volume of over 1,100 pages gives all kinds of particulars about newspapers of every country, with more or less fulness, arranged in such a way that the information is quite clear and easy of reference. The Advertisement Art Gallery, containing specimens of pictorial advertisements, and the excellent coloured facsimiles of the covers of many of the papers, makes interesting a work which is unquestionably of great utility to advertisers, business firms, journalists, and all who are in any way brought into contact with the press.

